# Supplementary material for: Mendelianization: Concentrating Polygenic Signal Into a Single Causal Locus
Source: Genet Epidemiol. 2026 Aug 14;50(6):e70053. doi: 10.1002/gepi.70053 (PMC13474947; doi:10.1002/gepi.70053)
Supplement: Supplementary file 1 [file GEPI-50-0-s001.pdf]

Supplementary Materials

A. Derivation of CCA Solution

Correlation and, likewise, the solution to Expression (1) is scale invariant, so we can impose the constraint $\alpha^\top \Sigma \alpha = 1$ and maximize the numerator $r_j \alpha$. We invoke the Cauchy-Schwarz inequality in the Σ-inner product:

$$r_j \alpha = \langle r_j \Sigma^{-1}, \alpha^\top \rangle_\Sigma \leq \| r_j \Sigma^{-1} \|_\Sigma \| \alpha^\top \|_\Sigma = \sqrt{r_j \Sigma^{-1} r_j^\top},$$

where the last equality follows because we imposed the constraint $\|\alpha^\top\|_\Sigma = \sqrt{\alpha^\top \Sigma \alpha} = 1$. The inequality becomes an equality when $\alpha = \frac{\Sigma^{-1} r_j^\top}{\sqrt{r_j \Sigma^{-1} r_j^\top}}$. Recall that the solution to Expression (2) is scale-invariant, so we set $\alpha = A r_j^\top$ with $A = \Sigma^{-1}$.

B. Finite m Assumptions

Assumption 5. *(Low-dimensional latent confounding) For a prespecified $m \in \mathbb{N}^+$, the confounding effects are contained in a subspace $U^m \subset \mathbb{R}^m$, and there exists an integer constant $r_0 \geq 0$ such that $\dim(U^m) \leq r_0$. We additionally assume that each vector of variant-outcome correlations admits a structural decomposition*

$$r_j^m = r_j^{m\parallel} + r_j^{m\perp},$$

where $r_j^{m\parallel} \in U^m$ is the confounding component and $r_j^{m\perp} \in (U^m)^{\perp_{A^m}}$ is the causal component, with orthogonality defined by $A^m = (\Sigma^m)^{-1}$.

Assumption 6. *(Outcome diversity) We have $\max_{k \notin \mathcal{S}_j} |\langle r_k^{m\perp}, r_j^{m\perp} \rangle_{A^m}| = 0$ for a prespecified m.*

Assumption 7. *(Signal floor) For a prespecified m, if $l \in \mathcal{S}_j$ causes $Y\alpha_j^m$, then there exists a constant $\beta_c > 0$ such that $\|r_l^{m\perp}\|_{A^m}^2 \geq \beta_c$. Moreover, there exists a constant $\beta_{on} > 0$ such that $\|r_l^{m\parallel}\|_{A^m}^2 \geq \beta_{on}$ for every $l \in \mathcal{S}_j$ that does not cause $Y\alpha_j^m$.*

Assumption 8. *(Isolation-spillover) If some $l \in \mathcal{S}_j$ causes $Y\alpha_j^m$, we have $\max_{k \notin \mathcal{S}_j} |\langle r_k^{m\parallel}, r_j^{m\parallel} \rangle_{A^m}| = 0$ for a prespecified m. On the other hand, if there does not exist $l \in \mathcal{S}_j$ that causes $Y\alpha_j^m$, then $\exists k \notin \mathcal{S}_j$ with $|\cos_{A^m}(r_j^{m\parallel}, r_k^{m\parallel})| \geq \gamma_j > 0$ and $\|r_k^{m\parallel}\|_{A^m}^2 \geq \beta_{off} > 0$ for a prespecified m.*

C. Pseudocode & Time Complexity Analysis

We present the pseudocode for Mendelianization in Algorithm 1. The algorithm first computes $\widehat{S}$ in Line 1, which estimates the realized conditional covariance $\Gamma^{(n)}(M)$ under Theorem 2 and targets Γ in the large-sample limit. Using this estimate, it then derives the raw canonical coefficients in Line 2 by solving Expression (5). These coefficients also form the basis of the Q statistics in Expression (3) that enable hypothesis testing; recall that each Q_j follows a χ_m^2 distribution under the null, so the algorithm can readily compute p-values in Line 4. Next, Mendelianization computes SoMs for lead variants that surpass a genome-wide significance threshold (e.g., 5×10^{-8}) in Line 5. Finally, the algorithm converts the raw coefficients into their interpretable form α' by applying Proposition 4 in Line 6. Thus, although the theory of Mendelianization is intricate, the resulting algorithm is remarkably straightforward.

Algorithm 1 Mendelianization

Input: multiple outcome z-statistics

Output: interpretable canonical coefficients α'; test statistics Q and p-values; Scores of Mendelianism $\mathcal{M}$

1: $\Gamma \leftarrow$ compute $\widehat{S}$ using z-statistics (Theorem 2)
2: $\alpha \leftarrow \Omega z^\top$
3: $Q \leftarrow \mathrm{diag}(z\alpha)$
4: $p \leftarrow$ right tail probability of each Q_j under χ_m^2
5: $\mathcal{M} \leftarrow$ compute SoMs (Equation (6); Theorem 1)
6: $\alpha' \leftarrow \alpha / \sqrt{\mathrm{diag}(\Omega)}$ (Proposition 4)

The algorithm also has a favorable time complexity. In particular, estimating the correlation matrix Γ described in Line 1 costs $O(qm^2)$ time. In Line 2, inverting Γ requires $O(m^3)$ time, and the matrix product $\Omega z^\top$ required to estimate α costs $O(m^2 q)$. Constructing all Q_j statistics and evaluating their p-values in Lines 3 and 4 adds $O(mq)$, and computing α' in Line 6 requires another $O(mq)$. For the SoM step, suppose there are s significant variants, and each constitutes its own tower to take the worst case. Computing each statistic $\mathcal{Z}_{k|j}$ requires $O(m)$ time for the numerator and $O(m^2)$ for the denominator in the definition of $\mathcal{Z}_{k|j}$. Moreover, the numerator must be evaluated genome-wide over q variants, so an upper bound on the total cost of SoM amounts to $O(s(mq + m^2))$. Summing these components, the overall complexity of Mendelianization is $O(m^3 + m^2 q + sqm)$, where the simplification uses $q \gg m$. Thus, the algorithm scales linearly with the number of variants and cubically with the number of outcome variables. In practice, Mendelianization completes within seconds and is at least three orders of magnitude faster than the competing methods.

D. Proofs

Theorem 1. *(Asymptotic Mendelianism) Consider Assumptions 1–4 and suppose Σ^m is positive definite for sufficiently large m. There exists a constant $c_j > 0$ such that $r_j^m \alpha_j^m \geq c_j$ for sufficiently large m and $\max_{k \notin \mathcal{S}_j} \left| r_k^m \alpha_j^m \right| \longrightarrow 0$ if and only if, for all sufficiently large m, at least one variant $l_m \in \mathcal{S}_j$ causes $Y\alpha_j^m$.*

Proof We first prove the forward direction by contrapositive. Suppose it is not the case that, for all sufficiently large m, there exists a variant $l \in \mathcal{S}_j$ that causes $Y\alpha_j^m$. Thus, for every $M_1 > 0$, there exists $m \geq M_1$ such that no variant $l \in \mathcal{S}_j$ causes $Y\alpha_j^m$. As a result, $j \in \mathcal{S}_j$ does not cause $Y\alpha_j^m$. We need to show $r_j^m \alpha_j^m$ is not bounded below by a positive constant for sufficiently large m or $\max_{k \notin \mathcal{S}_j} \left| r_k^m \alpha_j^m \right| \not\to 0$ (or both). We will show that $\max_{k \notin \mathcal{S}_j} \left| r_k^m \alpha_j^m \right| \not\to 0$.

Assumption 1 allows us to write:

$$\langle r_k^m, r_j^m \rangle_{A^m} = \langle r_k^{m\parallel}, r_j^{m\parallel} \rangle_{A^m} + \langle r_k^{m\perp}, r_j^{m\perp} \rangle_{A^m}. \tag{7}$$

Let $d_j = \sqrt{\beta_{\text{on}}}\sqrt{\beta_{\text{off}}}\gamma_j > 0$ and $\varepsilon_m = \max_{k \notin \mathcal{S}_j} \left| \langle r_k^{m\perp}, r_j^{m\perp} \rangle_{A^m} \right|$. Under Assumption 2, $\varepsilon_m \to 0$, so there exists $M_2 > 0$ such that for all $m \geq M_2$, $\varepsilon_m < d_j/2$ and Assumptions 3 and 4 apply.

Choose any $M_1 \geq M_2$. By the supposition above, there exists $m \geq M_1$ such that no variant $l \in \mathcal{S}_j$ causes $Y\alpha_j^m$. By Assumptions 3 and 4, there exists $k \notin \mathcal{S}_j$ such that

$$|\langle r_k^{m\parallel}, r_j^{m\parallel} \rangle_{A^m}| = \|r_j^{m\parallel}\|_{A^m} \|r_k^{m\parallel}\|_{A^m} |\cos_{A^m}(r_j^{m\parallel}, r_k^{m\parallel})| \geq \sqrt{\beta_{\text{on}}}\sqrt{\beta_{\text{off}}}\gamma_j = d_j.$$

The reverse triangle inequality allows us to write:

$$\begin{aligned} |\langle r_k^m, r_j^m \rangle_{A^m}| &= |\langle r_k^{m\parallel}, r_j^{m\parallel} \rangle_{A^m} + \langle r_k^{m\perp}, r_j^{m\perp} \rangle_{A^m}| \\ &\geq \Big| |\langle r_k^{m\parallel}, r_j^{m\parallel} \rangle_{A^m}| - |\langle r_k^{m\perp}, r_j^{m\perp} \rangle_{A^m}| \Big| \\ &\geq |\langle r_k^{m\parallel}, r_j^{m\parallel} \rangle_{A^m}| - |\langle r_k^{m\perp}, r_j^{m\perp} \rangle_{A^m}|. \end{aligned}$$

As a result,

$$|r_k^m \alpha_j^m| = |\langle r_k^m, r_j^m \rangle_{A^m}| \geq d_j - \varepsilon_m \geq \frac{d_j}{2} > 0.$$

Since $M_1 \geq M_2$ was arbitrary, for every sufficiently large M_1 there exists $m \geq M_1$ such that

$$\max_{k \notin \mathcal{S}_j} \left| r_k^m \alpha_j^m \right| \geq \frac{d_j}{2}.$$

Therefore, $\limsup_{m\to\infty} \max_{k \notin \mathcal{S}_j} \left| r_k^m \alpha_j^m \right| \geq \frac{d_j}{2} > 0$, so the off-locus associations cannot converge to zero. This completes the proof of sufficiency – i.e., asymptotic Mendelianism is sufficient to establish causality.

To prove the backward direction, assume that, for all sufficiently large m, there exists at least one variant $l_m \in \mathcal{S}_j$ that causes $Y\alpha_j^m$. Then, we can invoke Assumption 1 and write Equation (7) for any k. We have two situations:

1. If $k \notin \mathcal{S}_j$, then

$$\max_{k \notin \mathcal{S}_j} \left| \langle r_k^{m\perp}, r_j^{m\perp} \rangle_{A^m} \right| \to 0$$

   by Assumption 2. Moreover,

$$\max_{k \notin \mathcal{S}_j} \left| \langle r_k^{m\parallel}, r_j^{m\parallel} \rangle_{A^m} \right| \to 0$$

   by the first clause of Assumption 4. Hence,

$$\max_{k \notin \mathcal{S}_j} \left| \langle r_k^m, r_j^m \rangle_{A^m} \right| \to 0.$$

2. If $k \in \mathcal{S}_j$ and $k = j$, then we have two situations. If j causes $Y\alpha_j^m$, then the first clause of Assumption 3 allows us to write:

$$\langle r_j^m, r_j^m \rangle_{A^m} = |\langle r_j^m, r_j^m \rangle_{A^m}| = \|r_j^{m\perp}\|_{A^m}^2 + \|r_j^{m\parallel}\|_{A^m}^2 \geq \|r_j^{m\perp}\|_{A^m}^2 \geq \beta_{\text{c}} > 0,$$

   for sufficiently large m. On the other hand, if j does not cause $Y\alpha_j^m$, then the second clause of Assumption 3 allows us to write:

$$\langle r_j^m, r_j^m \rangle_{A^m} = \|r_j^{m\perp}\|_{A^m}^2 + \|r_j^{m\parallel}\|_{A^m}^2 \geq \|r_j^{m\parallel}\|_{A^m}^2 \geq \beta_{\text{on}} > 0,$$

   for sufficiently large m.

We conclude that there exists a constant $c_j = \min\{\beta_{\text{c}}, \beta_{\text{on}}\} > 0$ such that $r_j^m \alpha_j^m \geq c_j$ for sufficiently large m, and $\max_{k \notin \mathcal{S}_j} |r_k^m \alpha_j^m| \to 0$, which completes the proof of necessity – i.e., asymptotic Mendelianism is necessary to establish causality. □

Proposition 1. *(Perfect Mendelianism at finite m) Consider Assumptions 5–8 (Supplementary Materials B) with a prespecified m and suppose Σ^m is positive definite. We have $r_j^m \alpha_j^m > 0$ and $\max_{k \notin \mathcal{S}_j} \left| r_k^m \alpha_j^m \right| = 0$ (i.e., Mendelianism is perfectly achieved for $\mathcal{S}_j$ at m) if and only if at least one variant $l \in \mathcal{S}_j$ causes $Y\alpha_j^m$.*

Proof The proof almost exactly parallels that of Theorem 1, but we include it here for completeness. We first prove the forward direction by contrapositive. Suppose there does not exist a variant $l \in \mathcal{S}_j$ that causes $Y\alpha_j^m$. As a result, $j \in \mathcal{S}_j$ does not cause $Y\alpha_j^m$. We need to show $r_j^m \alpha_j^m = 0$ or $\max_{k \notin \mathcal{S}_j} |r_k^m \alpha_j^m| > 0$ (or both) for prespecified m. We will show that $\max_{k \notin \mathcal{S}_j} |r_k^m \alpha_j^m| > 0$.

Assumption 5 allows us to write:

$$\langle r_k^m, r_j^m \rangle_{A^m} = \langle r_k^{m\parallel}, r_j^{m\parallel} \rangle_{A^m} + \langle r_k^{m\perp}, r_j^{m\perp} \rangle_{A^m}. \tag{8}$$

By Assumptions 7 and 8, there exists $k \notin \mathcal{S}_j$ where:

$$\big|\langle r_k^{m\parallel}, r_j^{m\parallel} \rangle_{A^m}\big| \geq \|r_j^{m\parallel}\|_{A^m} \|r_k^{m\parallel}\|_{A^m} \gamma_j \geq \sqrt{\beta_{\text{on}}}\sqrt{\beta_{\text{off}}}\gamma_j.$$

The reverse triangle inequality allows us to write:

$$\begin{aligned}
&\big|\langle r_k^m, r_j^m \rangle_{A^m}\big| = \big|\langle r_k^{m\parallel}, r_j^{m\parallel} \rangle_{A^m} + \langle r_k^{m\perp}, r_j^{m\perp} \rangle_{A^m}\big| \\
\geq &\Big|\big|\langle r_k^{m\parallel}, r_j^{m\parallel} \rangle_{A^m}\big| - \big|\langle r_k^{m\perp}, r_j^{m\perp} \rangle_{A^m}\big|\Big| \geq \big|\langle r_k^{m\parallel}, r_j^{m\parallel} \rangle_{A^m}\big| - \big|\langle r_k^{m\perp}, r_j^{m\perp} \rangle_{A^m}\big|.
\end{aligned}$$

Thus, under Assumption 6, we have:

$$|r_k^m \alpha_j^m| = \big|\langle r_k^m, r_j^m \rangle_{A^m}\big| \geq \sqrt{\beta_{\text{on}}}\sqrt{\beta_{\text{off}}}\gamma_j > 0.$$

This completes the proof of sufficiency – i.e., perfect Mendelianism is sufficient to establish causality.

To prove the backward direction, assume that there exists at least one variant $l \in \mathcal{S}_j$ that causes $Y\alpha_j^m$. Then, we can invoke Assumption 5 and write Equation (8) for any k. We have two situations:

1. If $k \notin \mathcal{S}_j$, then $\max_{k \notin \mathcal{S}_j} |\langle r_k^{m\perp}, r_j^{m\perp} \rangle_{A^m}| = 0$ by Assumption 6. Moreover, $|\langle r_k^{m\parallel}, r_j^{m\parallel} \rangle_{A^m}| = 0$ by the first clause of Assumption 8. Hence, $\max_{k \notin \mathcal{S}_j} |\langle r_k^m, r_j^m \rangle_{A^m}| = 0$.
2. If $k \in \mathcal{S}_j$ and $k = j$, then we have two situations. If j causes $Y\alpha_j^m$, then the first clause of Assumption 7 allows us to write:

$$\langle r_j^m, r_j^m \rangle_{A^m} = |\langle r_j^m, r_j^m \rangle_{A^m}| = \|r_j^{m\perp}\|_{A^m}^2 + \|r_j^{m\parallel}\|_{A^m}^2 \geq \|r_j^{m\perp}\|_{A^m}^2 \geq \beta_{\text{c}} > 0.$$

On the other hand, if j does not cause $Y\alpha_j^m$, then the second clause of Assumption 7 allows us to write:

$$\langle r_j^m, r_j^m \rangle_{A^m} = \|r_j^{m\perp}\|_{A^m}^2 + \|r_j^{m\parallel}\|_{A^m}^2 \geq \|r_j^{m\parallel}\|_{A^m}^2 \geq \beta_{\text{on}} > 0.$$

We conclude that $r_j^m \alpha_j^m > 0$ and $\max_{k \notin \mathcal{S}_j} |r_k^m \alpha_j^m| = 0$, which completes the proof of necessity – i.e., perfect Mendelianism is necessary to establish causality. ☐

Proposition 2. *Assume i.i.d. samples across individuals, $V \perp\!\!\!\perp Y$, and $V \perp\!\!\!\perp M \mid Y$. Then $\mathbb{E}\big[z_{ja} z_{jb} \mid M\big] = \Gamma_{ab}^{(n)}(M)$, where $\Gamma_{ab}^{(n)}(M) := \frac{1}{\sqrt{n_a n_b}} \sum_{i \in S_a \cap S_b} \mathbb{E}(Y_{a,i} Y_{b,i} \mid M)$. Separately, without requiring $V \perp\!\!\!\perp Y$ or $V \perp\!\!\!\perp M \mid Y$, under i.i.d. missingness, with $p_a = P(M_a = 1) > 0$, $p_b = P(M_b = 1) > 0$, and $p_{ab} = P(M_a = 1, M_b = 1) > 0$, $\Gamma_{ab}^{(n)}(M) \xrightarrow{a.s.} \frac{p_{ab}}{\sqrt{p_a p_b}} \Sigma_{ab}^{\text{obs}}$.*

Proof Note that z-statistics are computed on observed samples, so

$$z_{ja} = \sqrt{n_a}\widehat{r}_{ja} = \frac{1}{\sqrt{n_a}} \sum_{i \in S_a} V_{ji} Y_{ai}, \qquad z_{jb} = \sqrt{n_b}\widehat{r}_{jb} = \frac{1}{\sqrt{n_b}} \sum_{k \in S_b} V_{jk} Y_{bk}.$$

Therefore

$$\mathbb{E}\big[z_{ja} z_{jb} \mid M\big] = \mathbb{E}\left[\frac{1}{\sqrt{n_a n_b}} \sum_{i \in S_a} \sum_{k \in S_b} V_{ji} Y_{ai} V_{jk} Y_{bk} \,\middle|\, M\right].$$

The cross-terms with $i \neq k$ vanish by i.i.d. sampling and the null assumptions. Indeed, $V \perp\!\!\!\perp Y$ and $V \perp\!\!\!\perp M \mid Y$ imply $V \perp\!\!\!\perp (Y, M)$ by contraction, and hence $V \perp\!\!\!\perp Y \mid M$ and $V \perp\!\!\!\perp M$. Since the genotypes are standardized, this gives $\mathbb{E}(V_{ji} \mid M) = 0$. Thus only

the terms with $i = k \in S_a \cap S_b$ remain:

$$\mathbb{E}\big[z_{ja} z_{jb} \mid M\big] = \frac{1}{\sqrt{n_a n_b}} \sum_{i \in S_a \cap S_b} \mathbb{E}\Big[V_{ji}^2 Y_{ai} Y_{bi} \,\Big|\, M\Big].$$

Using $V \perp\!\!\!\perp Y \mid M$ and $V \perp\!\!\!\perp M$,

$$\mathbb{E}\Big[V_{ji}^2 Y_{ai} Y_{bi} \,\Big|\, M\Big] = \mathbb{E}\Big(V_{ji}^2 \mid M\Big)\, \mathbb{E}(Y_{ai} Y_{bi} \mid M) = \mathbb{E}(Y_{ai} Y_{bi} \mid M),$$

because $\mathbb{E}(V_{ji}^2 \mid M) = \mathbb{E}(V_{ji}^2) = 1$. Hence

$$\mathbb{E}\big[z_{ja} z_{jb} \mid M\big] = \frac{1}{\sqrt{n_a n_b}} \sum_{i \in S_a \cap S_b} \mathbb{E}(Y_{ai} Y_{bi} \mid M) = \Gamma_{ab}^{(n)}(M).$$

It remains to identify the large-sample limit. Write M_i for the missingness pattern of individual i. Under i.i.d. missingness, $\mathbb{E}(Y_{ai} Y_{bi} \mid M) = \mathbb{E}(Y_{ai} Y_{bi} \mid M_i)$, and

$$\Gamma_{ab}^{(n)}(M) = \frac{n_{ab}/n}{\sqrt{(n_a/n)(n_b/n)}} \left\{ \frac{1}{n_{ab}} \sum_{i \in S_a \cap S_b} \mathbb{E}(Y_{ai} Y_{bi} \mid M_i) \right\}.$$

By the strong law of large numbers, $n_a/n \to p_a$, $n_b/n \to p_b$, and $n_{ab}/n \to p_{ab}$ almost surely. Also,

$$\frac{1}{n_{ab}} \sum_{i \in S_a \cap S_b} \mathbb{E}(Y_{ai} Y_{bi} \mid M_i) \xrightarrow{a.s.} \mathbb{E}(Y_a Y_b \mid M_a = 1, M_b = 1) = \Sigma_{ab}^{\text{obs}}.$$

Therefore $\Gamma_{ab}^{(n)}(M) \xrightarrow{a.s.} \frac{p_{ab}}{\sqrt{p_a p_b}} \Sigma_{ab}^{\text{obs}}$. □

Proposition 3. *Assume i.i.d. samples across individuals, $p_a > 0$, $p_b > 0$, $p_{ab} > 0$, and the corresponding conditional product moments are uniformly integrable. Then $\mathbb{E}\Big(\frac{1}{n}\sum_t Y'_{a,t} Y'_{b,t} \mid M\Big) = \Gamma_{ab}^{(n)}(M) \xrightarrow{a.s.} \Gamma_{ab} = \frac{p_{ab}}{\sqrt{p_a p_b}} \Sigma_{ab}^{\text{obs}}$ and $\mathbb{E}\Big(\frac{1}{n}\sum_t Y'_{a,t} Y'_{b,t}\Big) \to \Gamma_{ab} = \frac{p_{ab}}{\sqrt{p_a p_b}} \Sigma_{ab}^{\text{obs}}$ as $n \to \infty$.*

Proof For the first statement, using $Y'_{a,t} = \sqrt{n/n_a}\, M_{a,t} Y_{a,t}$, we have

$$\mathbb{E}\left(\frac{1}{n}\sum_t Y'_{a,t} Y'_{b,t} \mid M\right) = \mathbb{E}\left(\frac{1}{\sqrt{n_a n_b}} \sum_t M_{a,t} M_{b,t} Y_{a,t} Y_{b,t} \mid M\right) = \frac{1}{\sqrt{n_a n_b}} \sum_{t \in S_a \cap S_b} \mathbb{E}(Y_{a,t} Y_{b,t} \mid M) = \Gamma_{ab}^{(n)}(M).$$

Under i.i.d. missingness,

$$\Gamma_{ab}^{(n)}(M) = \frac{n_{ab}/n}{\sqrt{(n_a/n)(n_b/n)}} \left\{ \frac{1}{n_{ab}} \sum_{t \in S_a \cap S_b} \mathbb{E}(Y_{a,t} Y_{b,t} \mid M_t) \right\}.$$

Since $p_a > 0$, $p_b > 0$, and $p_{ab} > 0$, the strong law of large numbers gives $n_a/n \to p_a$, $n_b/n \to p_b$, and $n_{ab}/n \to p_{ab}$ almost surely, and the pairwise-observed average satisfies

$$\frac{1}{n_{ab}} \sum_{t \in S_a \cap S_b} \mathbb{E}(Y_{a,t} Y_{b,t} \mid M_t) \xrightarrow{a.s.} \mathbb{E}(Y_a Y_b \mid M_a = 1, M_b = 1) = \Sigma_{ab}^{\text{obs}}.$$

Therefore $\Gamma_{ab}^{(n)}(M) \xrightarrow{a.s.} \frac{p_{ab}}{\sqrt{p_a p_b}} \Sigma_{ab}^{\text{obs}} = \Gamma_{ab}$.

For the second statement, by the law of total expectation,

$$\mathbb{E}\left(\frac{1}{n}\sum_t Y'_{a,t} Y'_{b,t}\right) = \mathbb{E}_M\left\{\mathbb{E}\left(\frac{1}{n}\sum_t Y'_{a,t} Y'_{b,t} \mid M\right)\right\} = \mathbb{E}_M\{\Gamma_{ab}^{(n)}(M)\}.$$

Since $\Gamma_{ab}^{(n)}(M) \to \Gamma_{ab}$ almost surely, and the corresponding conditional product moments are uniformly integrable, it follows that, $\mathbb{E}_M\{\Gamma_{ab}^{(n)}(M)\} \to \Gamma_{ab} = \frac{p_{ab}}{\sqrt{p_a p_b}} \Sigma_{ab}^{\text{obs}}$. Hence $\mathbb{E}\Big(\frac{1}{n}\sum_t Y'_{a,t} Y'_{b,t}\Big) \to \Gamma_{ab}$. □

Theorem 2. *For fixed outcomes a and b, consider the estimator $\widehat{S}_{ab,n} = \frac{1}{q_n}\sum_{j=1}^{q_n} z_{ja,n} z_{jb,n}$, where $q_n \to \infty$ as $n \to \infty$. Let G_n index the variants that are independent of Y, and let B_n index the variants that are dependent on Y. Define $X_{j,n} = z_{ja,n} z_{jb,n}$. Assume the conditions of the second part of Proposition 2 hold and further assume:*

1. *(Asymptotic null centering) There exists a sequence $\eta_n \to 0$ such that $\sup_{j \in G_n} \Big|\mathbb{E}(X_{j,n} \mid M) - \Gamma_{ab}^{(n)}(M)\Big| \le \eta_n$.*
2. *(Vanishing aggregate contamination) We have $\frac{1}{q_n}\sum_{j \in B_n} \Big|\mathbb{E}(X_{j,n} \mid M) - \Gamma_{ab}^{(n)}(M)\Big| \to 0$.*

3. *(Local dependence) Conditional on M, the variables $\{X_{j,n}\}_{j=1}^{q_n}$ admit an undirected dependency graph with maximum degree D_n, and $\frac{D_n+1}{q_n^2}\sum_{j=1}^{q_n}\mathrm{Var}(X_{j,n}\mid M)\to 0$.*

Then $\widehat{S}_{ab,n}\xrightarrow{p}\Gamma_{ab}$.

Proof Let $X_{j,n}=z_{ja,n}z_{jb,n}$. Consider the unnormalized sum $N_{ab,n}=\sum_{j=1}^{q_n}X_{j,n}$, so that $\widehat{S}_{ab,n}=N_{ab,n}/q_n$. Write:

$$\sum_{j=1}^{q_n}\left(X_{j,n}-\Gamma_{ab}^{(n)}(M)\right)=\underbrace{\sum_{j\in G_n}\left(X_{j,n}-\Gamma_{ab}^{(n)}(M)\right)}_{N_G}+\underbrace{\sum_{j\in B_n}\left(X_{j,n}-\Gamma_{ab}^{(n)}(M)\right)}_{N_B}.$$

By Condition 1, we have

$$\begin{aligned}|\mathbb{E}(N_G\mid M)|&=\left|\sum_{j\in G_n}\left\{\mathbb{E}(X_{j,n}\mid M)-\Gamma_{ab}^{(n)}(M)\right\}\right|\\&\le\sum_{j\in G_n}\left|\mathbb{E}(X_{j,n}\mid M)-\Gamma_{ab}^{(n)}(M)\right|\\&\le\eta_n|G_n|.\end{aligned}$$

We can also write:

$$|\mathbb{E}(N_B\mid M)|\le\sum_{j\in B_n}\left|\mathbb{E}(X_{j,n}\mid M)-\Gamma_{ab}^{(n)}(M)\right|.$$

The inequality follows by the triangle inequality. Thus, by Conditions 1 and 2,

$$\begin{aligned}\left|\mathbb{E}(\widehat{S}_{ab,n}\mid M)-\Gamma_{ab}^{(n)}(M)\right|&\le\frac{\eta_n|G_n|}{q_n}+\frac{1}{q_n}\sum_{j\in B_n}\left|\mathbb{E}(X_{j,n}\mid M)-\Gamma_{ab}^{(n)}(M)\right|\\&\le\eta_n+\frac{1}{q_n}\sum_{j\in B_n}\left|\mathbb{E}(X_{j,n}\mid M)-\Gamma_{ab}^{(n)}(M)\right|\to 0.\end{aligned}$$

Let $v_{j,n}=\mathrm{Var}(X_{j,n}\mid M)$, and let E_n denote the edge set of the dependency graph in Condition 3. We can write:

$$\begin{aligned}\mathrm{Var}(\widehat{S}_{ab,n}\mid M)&=\frac{1}{q_n^2}\sum_{j=1}^{q_n}v_{j,n}+\frac{2}{q_n^2}\sum_{(j,k)\in E_n}\mathrm{cov}(X_{j,n},X_{k,n}\mid M)\\&\le\frac{1}{q_n^2}\sum_{j=1}^{q_n}v_{j,n}+\frac{2}{q_n^2}\sum_{(j,k)\in E_n}\sqrt{v_{j,n}v_{k,n}}\\&\le\frac{1}{q_n^2}\sum_{j=1}^{q_n}v_{j,n}+\frac{1}{q_n^2}\sum_{(j,k)\in E_n}(v_{j,n}+v_{k,n})\\&\le\frac{D_n+1}{q_n^2}\sum_{j=1}^{q_n}v_{j,n}.\end{aligned}$$

The first inequality follows from the conditional Cauchy–Schwarz inequality, the second from $2\sqrt{v_{j,n}v_{k,n}}\le v_{j,n}+v_{k,n}$, and the final inequality follows because each vertex has degree at most D_n. Therefore, Condition 3 implies $\mathrm{Var}(\widehat{S}_{ab,n}\mid M)\to 0$.

Let $\mu_n=\mathbb{E}(\widehat{S}_{ab,n}\mid M)$. From the expectation calculation above, $\left|\mu_n-\Gamma_{ab}^{(n)}(M)\right|\to 0$. For any $\varepsilon>0$, we therefore have

$$\varepsilon-\left|\mu_n-\Gamma_{ab}^{(n)}(M)\right|\ge\frac{\varepsilon}{2}$$

for sufficiently large n. We invoke the triangle inequality to write:

$$\begin{aligned}&\mathbb{P}_{|M}\left(\left|\widehat{S}_{ab,n}-\Gamma_{ab}^{(n)}(M)\right|>\varepsilon\right)\\&\quad=\mathbb{P}_{|M}\left(\left|(\widehat{S}_{ab,n}-\mu_n)+(\mu_n-\Gamma_{ab}^{(n)}(M))\right|>\varepsilon\right)\\&\quad\le\mathbb{P}_{|M}\left(|\widehat{S}_{ab,n}-\mu_n|>\varepsilon-|\mu_n-\Gamma_{ab}^{(n)}(M)|\right)\\&\quad\le\mathbb{P}_{|M}\left(|\widehat{S}_{ab,n}-\mu_n|>\frac{\varepsilon}{2}\right),\end{aligned}$$

where we have moved the conditioning notation to the subscripts to avoid confusion with absolute value notation. Chebyshev's inequality gives

$$\mathbb{P}_{|M}\left(|\widehat{S}_{ab,n}-\mu_n|>\frac{\varepsilon}{2}\right)\le\frac{4\mathrm{Var}(\widehat{S}_{ab,n}\mid M)}{\varepsilon^2}\to 0.$$

Therefore, $\widehat{S}_{ab,n}-\Gamma_{ab}^{(n)}(M)\xrightarrow{p}0$ conditional on M, and hence also unconditionally by dominated convergence.

Finally, Proposition 2 gives $\Gamma_{ab}^{(n)}(M) \overset{a.s.}{\to} \Gamma_{ab}$ as $n \to \infty$. Thus,

$$\widehat{S}_{ab,n} - \Gamma_{ab} = \left\{\widehat{S}_{ab,n} - \Gamma_{ab}^{(n)}(M)\right\} + \left\{\Gamma_{ab}^{(n)}(M) - \Gamma_{ab}\right\} \overset{p}{\to} 0.$$

This proves $\widehat{S}_{ab,n} \overset{p}{\to} \Gamma_{ab}$. □

E. Additional Experimental Results

Simulations

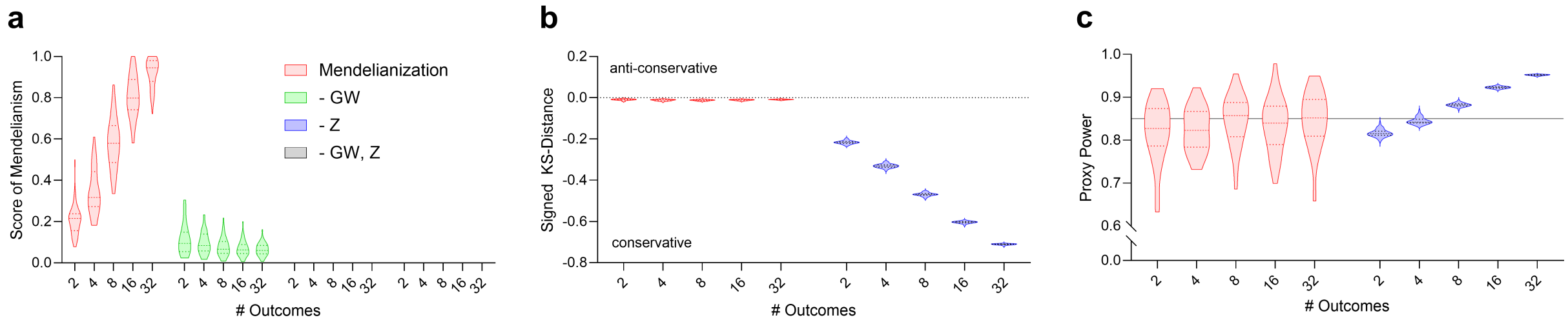

Supplementary Fig. 1. Ablation results. "-GW" denotes not applying each learned outcome genome-wide, "-Z" denotes estimating Γ from β coefficients instead of z-statistics, and "-GW, Z" denotes using both modifications. (a) Misapplying the CCA-derived outcomes (-GW) or using β coefficients in place of z-statistics (-Z) reduced the SoM to essentially zero; for two of the ablations, the SoM violin plots collapse at the bottom and are therefore not visible. (b) Using β coefficients produced highly conservative p-values. (c) The corresponding p-value histogram was strongly U-shaped, indicating severe miscalibration and yielding highly unstable power across outcome dimensions. Despite these deficiencies, the -GW and -Z variants still achieved near-perfect true positive rates and completed in under one second, comparable to Mendelianization.

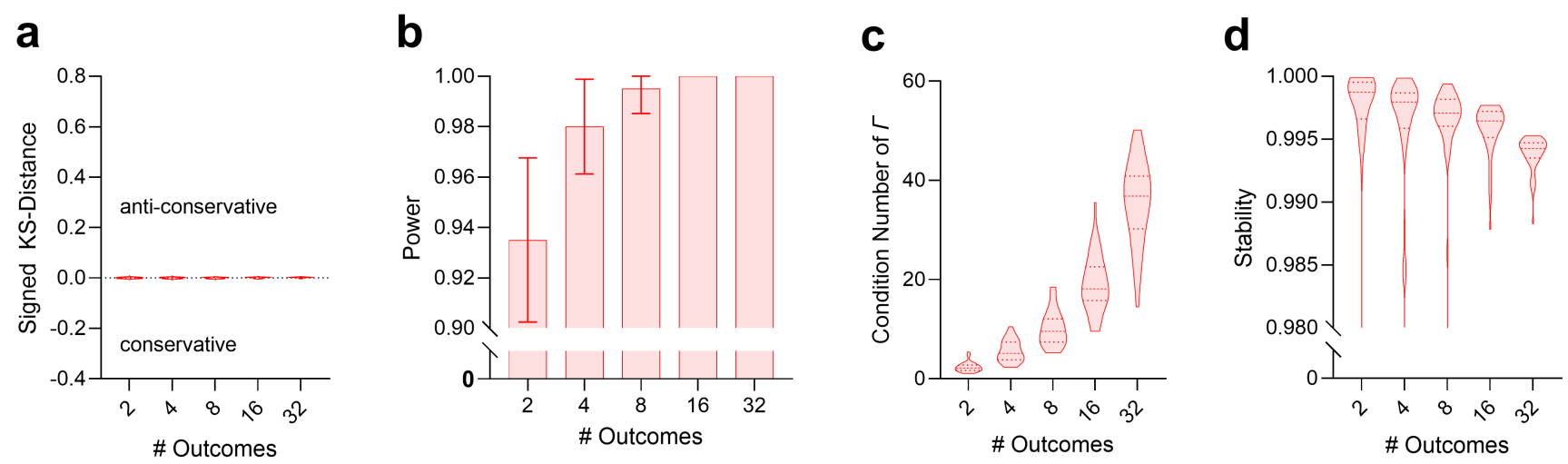

Supplementary Fig. 2. Null-simulation, power, conditioning, and stability results. (a) In global-null simulations, where no variant causally affected any outcome, Mendelianization produced p-values that closely followed the expected uniform distribution. (b) In simulations with known causal variants, Mendelianization achieved high empirical power at the genome-wide threshold, with power increasing as the number of outcomes grew. (c) The condition number of Γ increased with the number of outcomes, but never exceeded 51, indicating numerical stability across the simulated settings. (d) Composite stability remained high across all outcome dimensions, with Γ-weighted cosine similarities usually above 0.99. Thus, the increasing condition number did not translate into instability of the learned composites.

Simulations with Confounding

To evaluate robustness under latent confounding, we repeated the same semi-synthetic simulation pipeline after adding a sparse low-rank confounded component to the latent traits. Let $\widetilde{Y}_{\text{causal}} \in \mathbb{R}^{n\times 50}$ denote the same causal latent-trait matrix generated in the non-confounded simulation from the five causal variants. In each replicate, we additionally sampled 10 LD blocks and selected one tagging variant from each block. These tagging variants were assigned to $r_0 = 2$ shared latent confounding factors. For each factor g, we formed a confounder score by taking a randomly signed sum of the genotypes of the tagging variants assigned to that factor,

$$U_{ig} = \sum_{b\in\mathcal{B}_g} s_b V_{i,t_b}, \qquad s_b \in \{-1, 1\},$$

where t_b is the tagging variant selected from confounded block b, and then standardized each column of U. We then drew a random loading matrix $B_{\text{conf}} \in \mathbb{R}^{2\times 50}$ with independent standard normal entries and generated a raw confounded latent-trait component $\widetilde{Y}^{\text{raw}}_{\text{conf}} = UB_{\text{conf}}$. Thus, only two latent confounding factors affected all 50 latent traits, but they did so through heterogeneous trait-specific loadings. To make the confounded component comparable in magnitude to the causal component, we rescaled it as

$$\widetilde{Y}_{\text{conf}} = \frac{\text{sd}\{\text{vec}(\widetilde{Y}_{\text{causal}})\}}{\text{sd}\{\text{vec}(\widetilde{Y}^{\text{raw}}_{\text{conf}})\}}\widetilde{Y}^{\text{raw}}_{\text{conf}},$$

and then generated the final latent traits as $\widetilde{Y} = \widetilde{Y}_{\text{causal}} + \widetilde{Y}_{\text{conf}}$. The observed outcomes and summary statistics were then generated from $\widetilde{Y}$ using the same outcome-mixing, sample-size, and blockwise bootstrapping procedure as in the non-confounded simulations.

This construction was designed to mirror the confounding assumptions in the theory. The rank-two matrix UB_{conf} corresponds to the low-dimensional latent confounding condition in Assumption 1. The reuse of the same two confounding directions across

multiple LD blocks enforces the isolation–spillover behavior in Assumption 4: a purely confounded signal should tend to reappear at other blocks sharing the same latent confounding direction, rather than producing an isolated single-locus tower. Finally, scaling $\widetilde{Y}_{\mathrm{conf}}$ to match the empirical scale of $\widetilde{Y}_{\mathrm{causal}}$ ensures that the confounded component is non-negligible, creating a meaningful stress test rather than a vanishing perturbation.

We summarize the confounded synthetic-data results in Supplementary Figure 3. Overall, Mendelianization produced results that were qualitatively identical to the corresponding no-confounding experiments, supporting the theoretical prediction that low-dimensional spillover confounding should not by itself create isolated Mendelianized towers. The ablation results showed the same pattern (Supplementary Figure 4); Mendelianization was again the only method that simultaneously achieved increasing SoM with more outcomes, maintained Type I error control, and preserved stable proxy power.

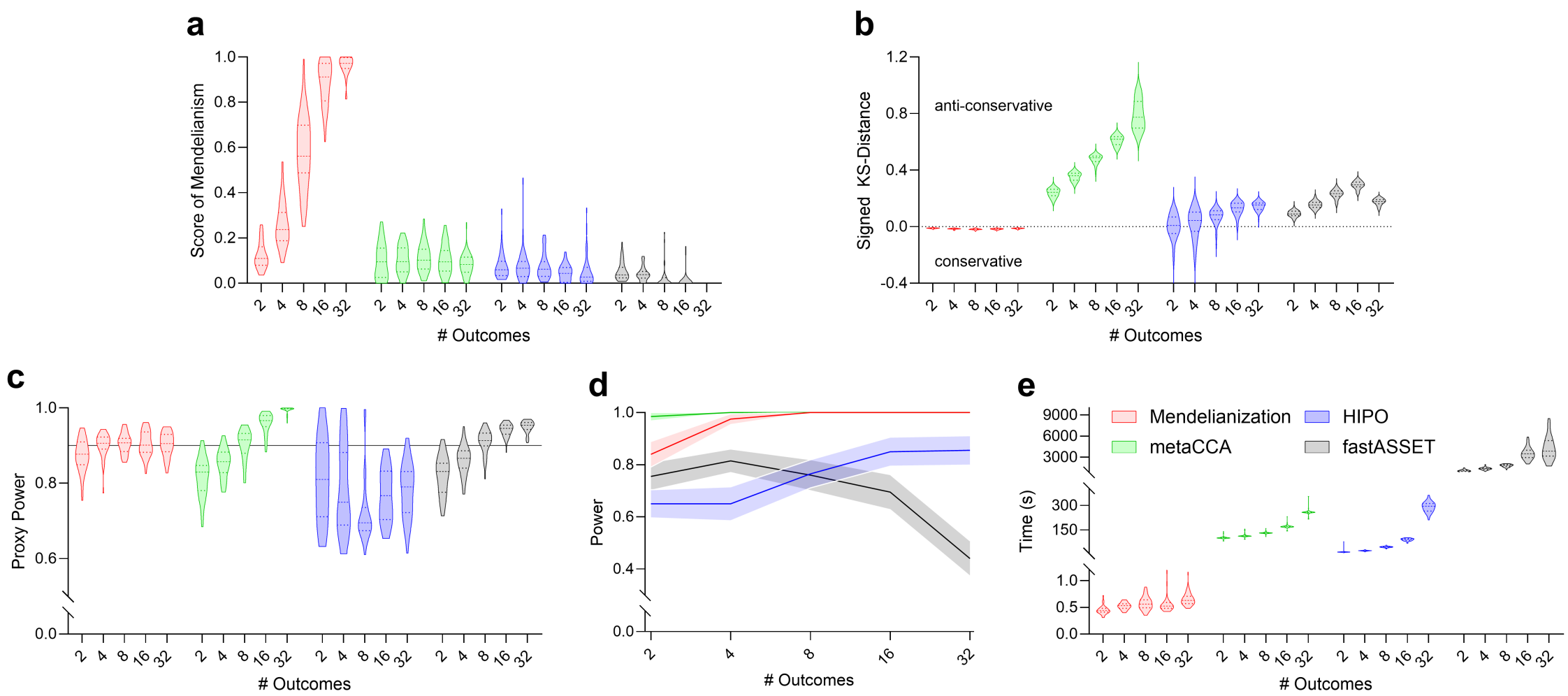

Supplementary Fig. 3. Synthetic-data results with latent confounding. The same simulation design as in Figure 2 was repeated after adding sparse low-rank latent confounding shared across multiple LD blocks. Results closely matched the no-confounding setting.

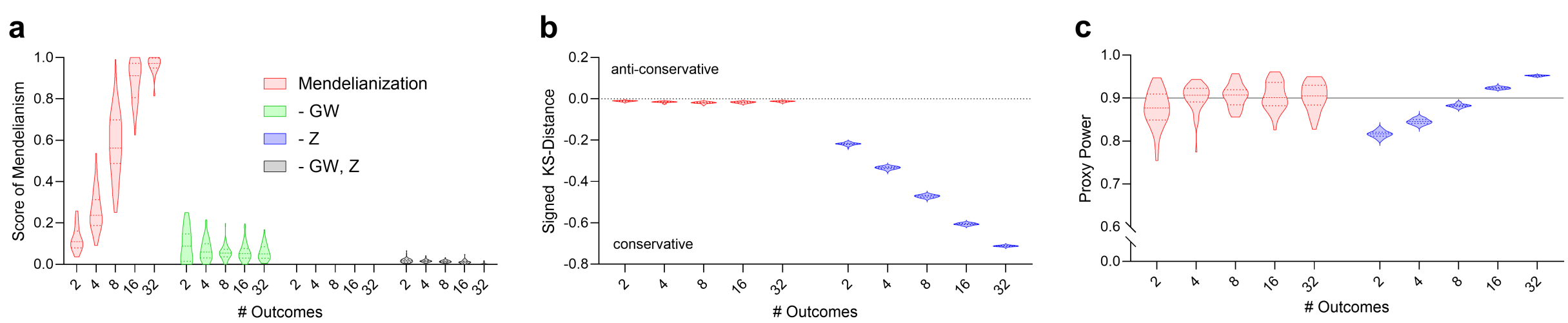

Supplementary Fig. 4. Ablation results with latent confounding. Ablation analyses under the confounded synthetic-data setting qualitatively matched the corresponding no-confounding results in Supplementary Figure 1.

Depression and Generalized Anxiety

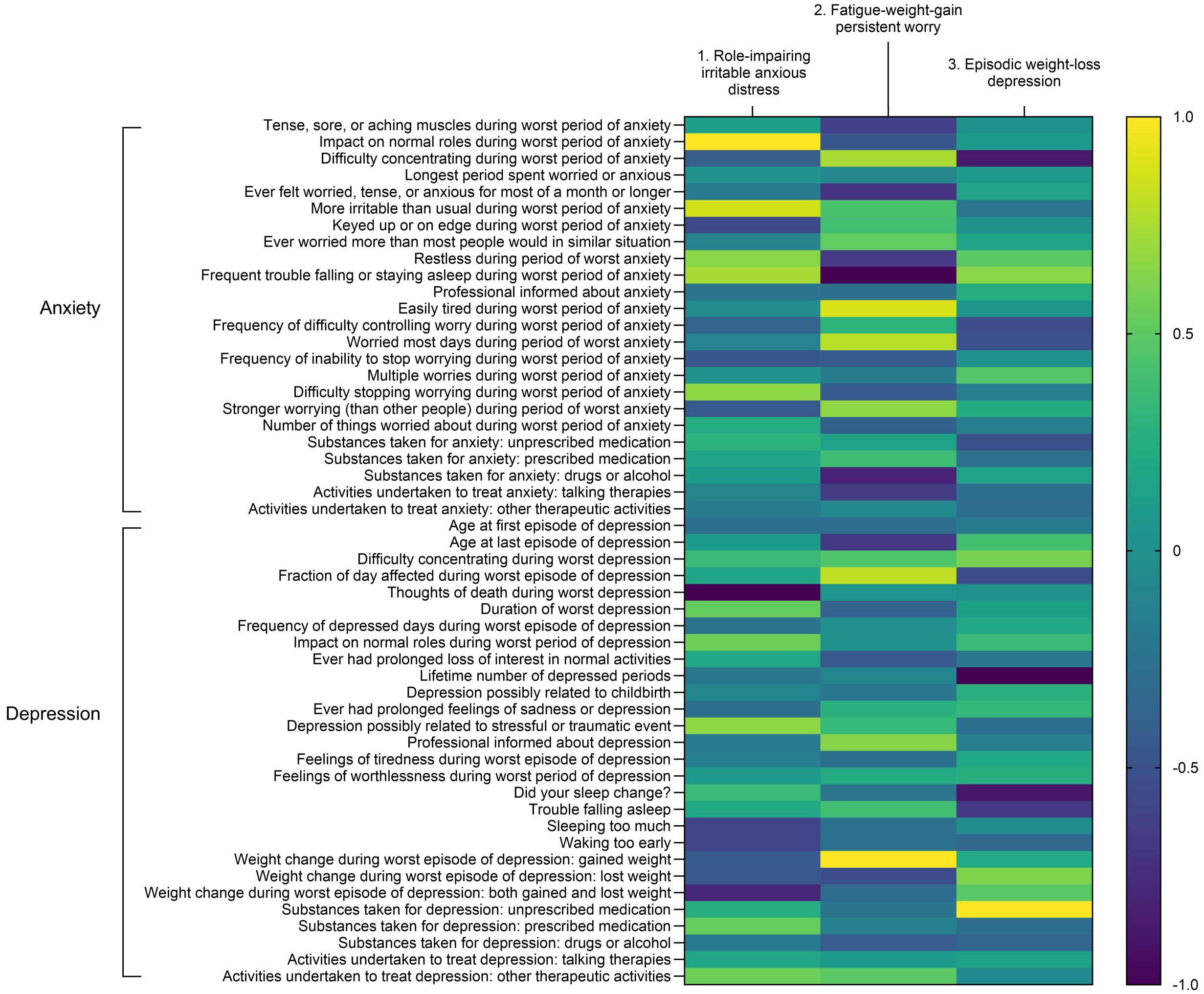

Supplementary Fig. 5. Heatmap of interpretable α' coefficients for lead variants in depression and anxiety. Each column displays the coefficients for a lead variant. The coefficients are interpreted as an allele-oriented symptom profile: positive entries indicate unique symptom components positively associated with higher dosage of the allele used to orient the input z-statistics, whereas negative entries indicate unique symptom components negatively associated with higher dosage of that same allele. Thus, the signs should be read jointly within a column as a contrast among residualized symptom components. To emphasize this contrast structure, positive and negative coefficients were normalized separately within each column to the range $[-1, 1]$. The resulting profiles were labeled as: (i) role-impairing irritable anxious distress; (ii) fatigue-weight-gain persistent worry; and (iii) episodic weight-loss depression. These profiles illustrate distinct locus-specific dimensions of depression and anxiety, underscoring the heterogeneity of these disorders.

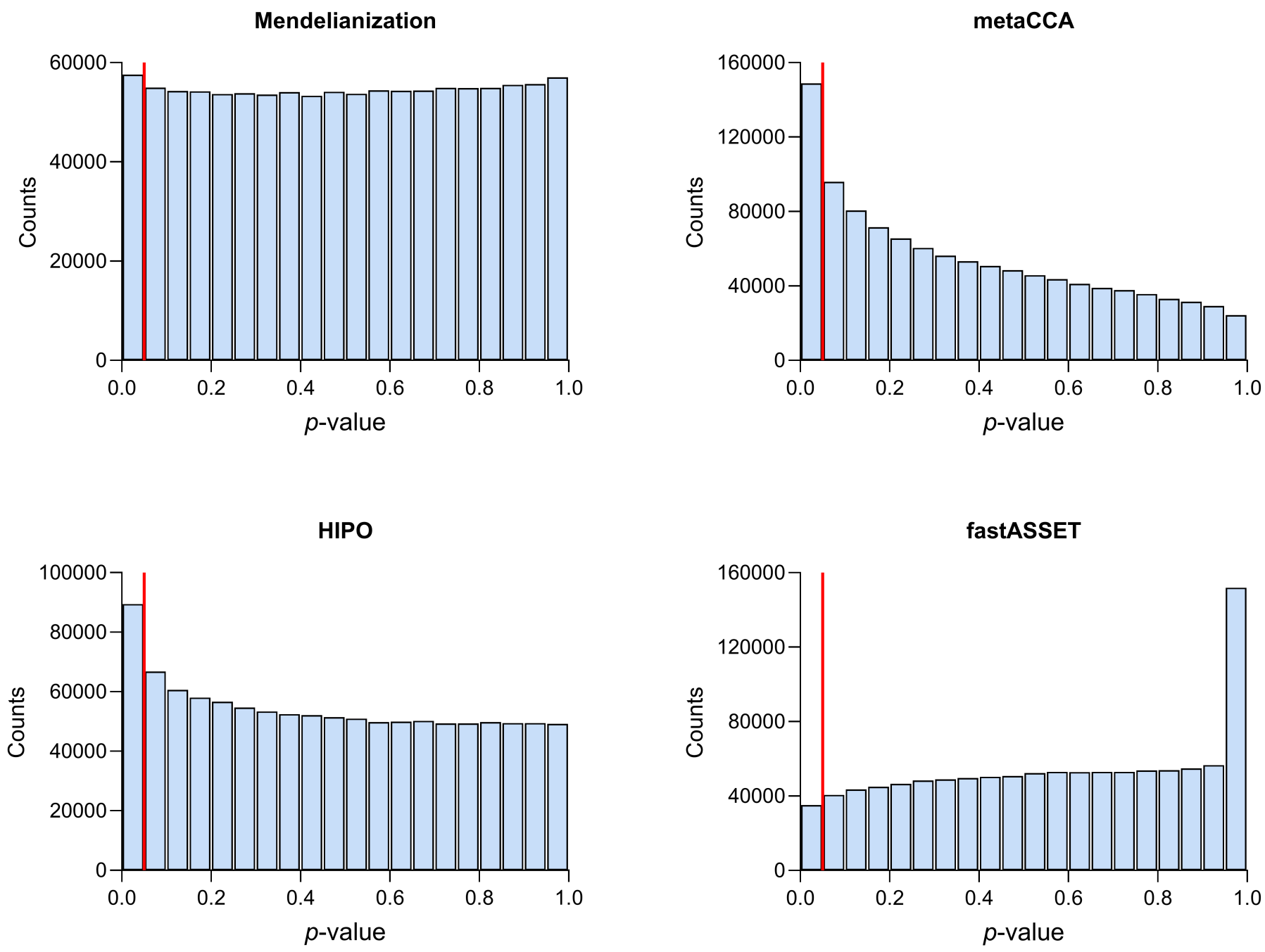

Supplementary Fig. 6. Histograms of p-values across all variants. Mendelianization was the only method to produce a near-uniform distribution for variants associated with p-values above 0.05 (red line). By contrast, metaCCA and HIPO yielded anti-conservative distributions with shrunken p-values, whereas fastASSET was overly conservative, producing p-values near one for many variants.

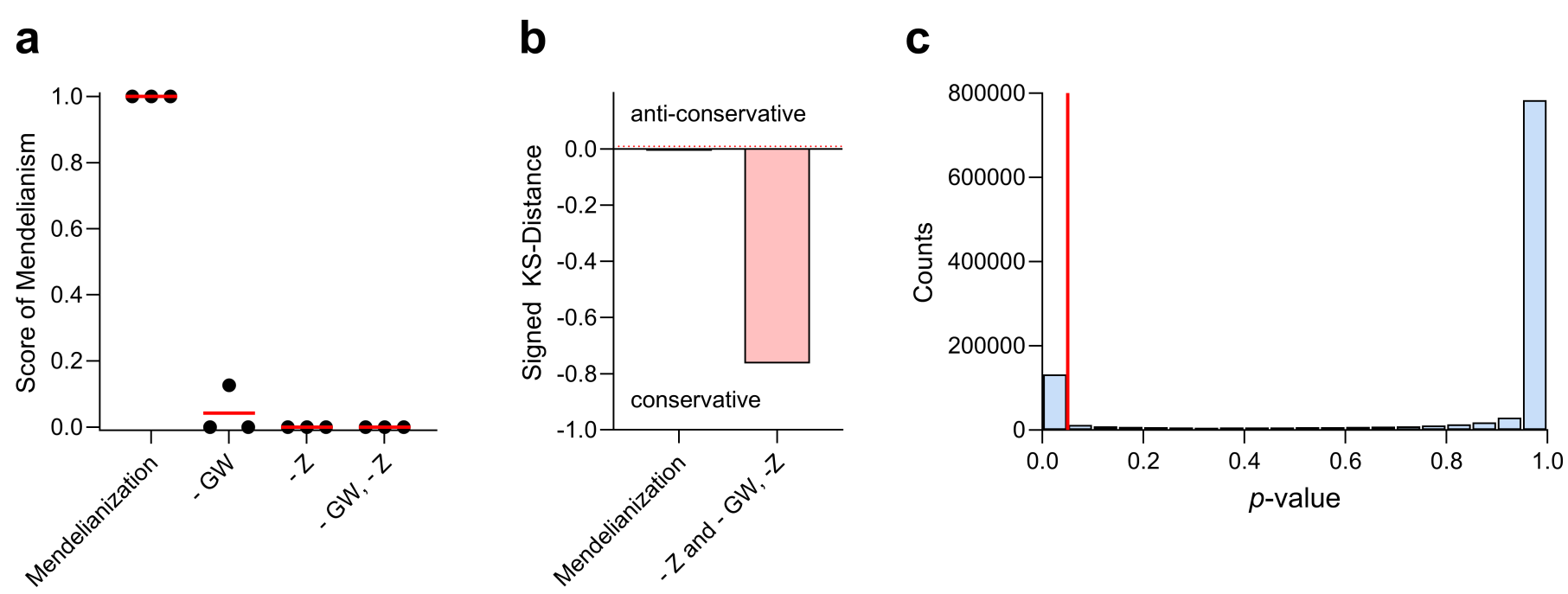

Supplementary Fig. 7. Ablation results. (a) Misapplying the learned outcomes or using β coefficients in place of z-statistics (or both) decreased the SoM score to near zero. (b) The histogram obtained by β coefficients was very conservative. (c) Inspection of the histogram derived from the β coefficient estimates revealed highly miscalibrated p-values following a U-shape curve.

Alcohol Use Disorder

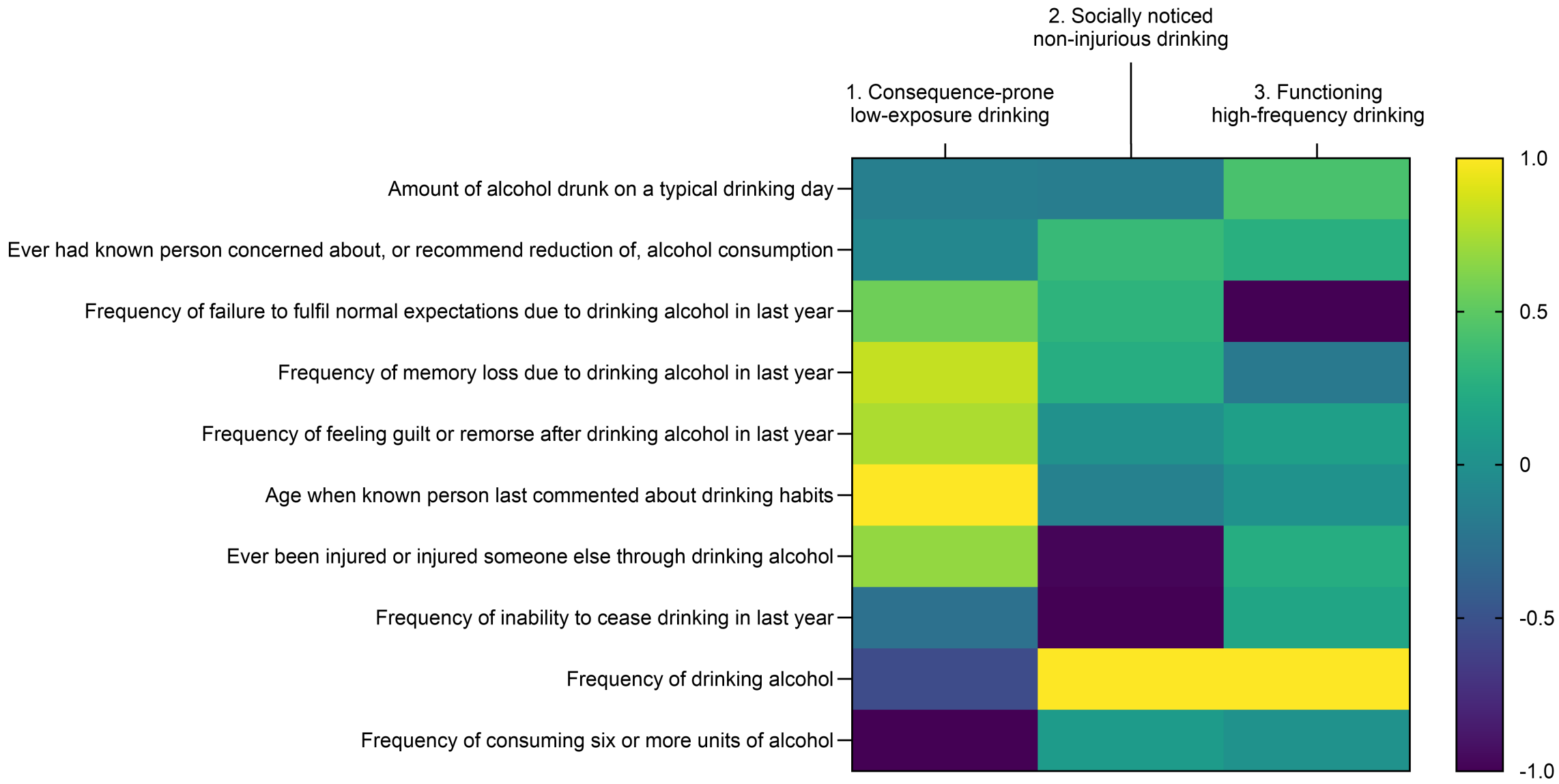

Supplementary Fig. 8. Heatmap of α' coefficients for lead variants in alcohol use disorder. As in Supplementary Figure 5, each column should be read as a signed, allele-oriented profile of unique AUDIT item components. Positive entries identify AUDIT components associated with higher effect-allele dosage at the lead variant, whereas negative entries identify components associated in the opposite direction. The labels summarize the overall profile of each column. The coefficient patterns resolve three AUD profiles: (i) consequence-prone low-exposure drinking; (ii) socially noticed regular drinking without severe complications; and (iii) high-frequency drinking with preserved role function.

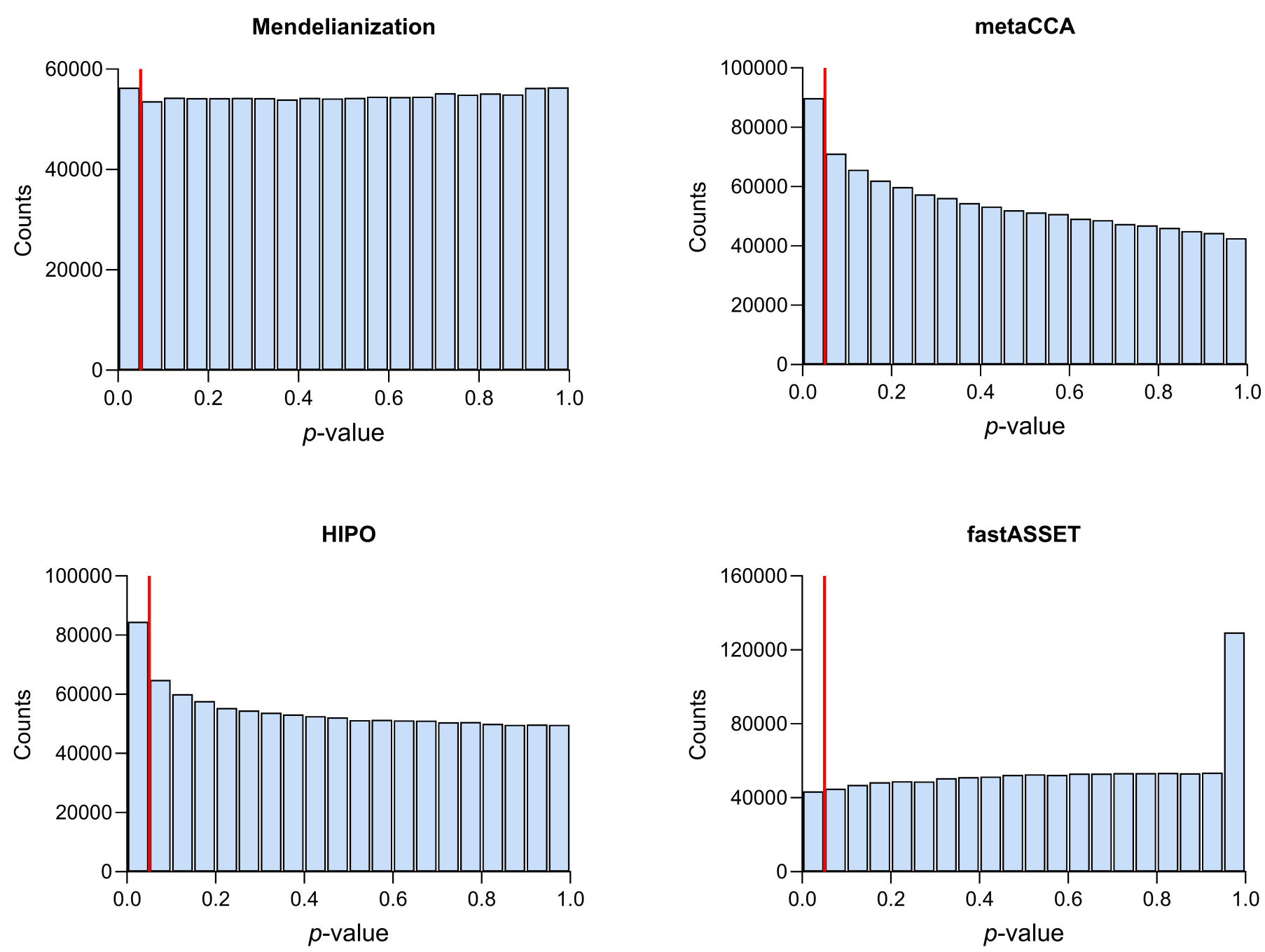

Supplementary Fig. 9. Histograms of p-values across all variants. Mendelianization was again the only method to produce a near-uniform distribution on $[0.05, 1]$.

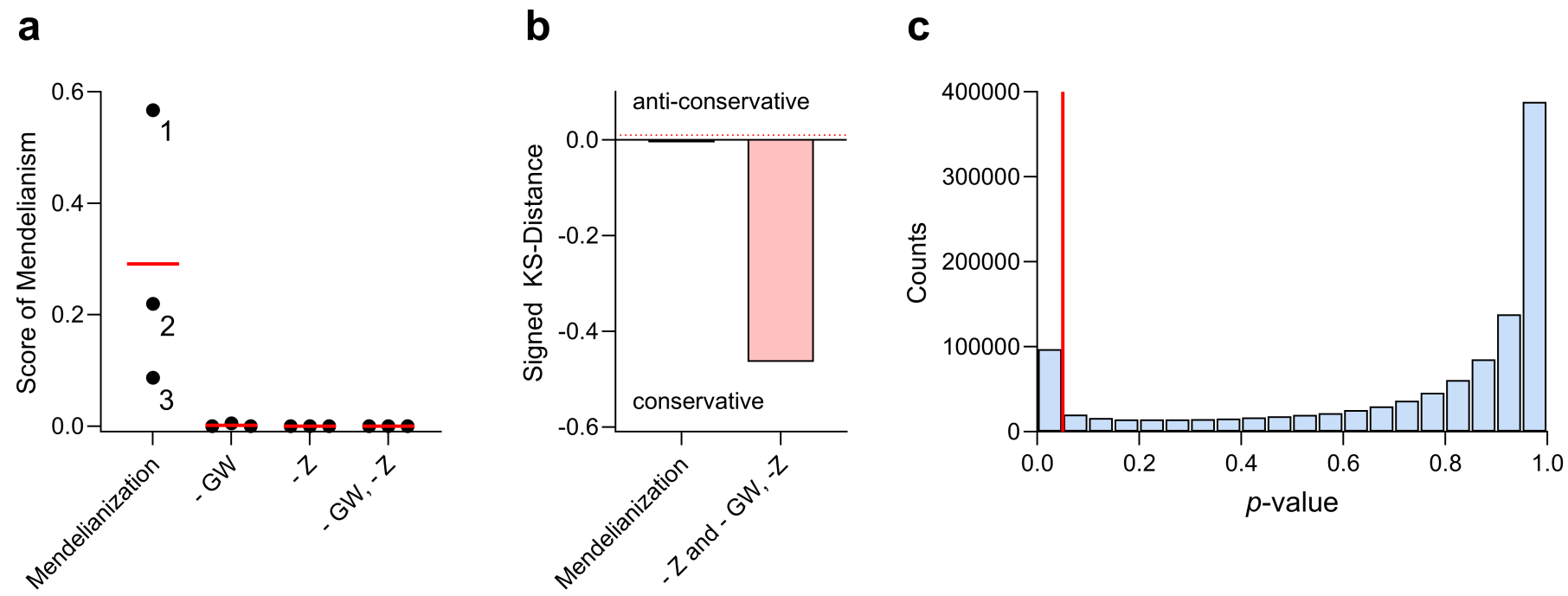

Supplementary Fig. 10. Ablation results. (a) Mendelianization again achieved the highest SoMs, and (b, c) estimating Γ from β coefficients yielded highly miscalibrated p-values.
